# T-cells in human trigeminal ganglia express canonical tissue-resident memory T-cell markers

**DOI:** 10.1186/s12974-022-02611-x

**Published:** 2022-10-06

**Authors:** Peter-Paul A. Unger, Anna E. Oja, Tamana Khemai-Mehraban, Werner J. D. Ouwendijk, Pleun Hombrink, Georges M. G. M. Verjans

**Affiliations:** 1 Department of Viroscience, Erasmus MC, Molewaterplein 40, 3015 GD Rotterdam, The Netherlands; 2 Department of Hematopoiesis, Sanquin Research and Landsteiner Laboratory, Amsterdam UMC, University of Amsterdam, Amsterdam, The Netherlands

**Keywords:** Human, Trigeminal ganglion, Normal-appearing white matter, Tissue-resident memory T-cells and herpes simplex virus

## Abstract

**Background:**

Trigeminal ganglia (TG) neurons are the main site of lifelong latent herpes simplex virus type 1 (HSV-1) infection. T-cells in ganglia contribute to long-term control of latent HSV-1 infection, but it is unclear whether these cells are bona fide tissue-resident memory T-cells (T_RM_). We optimized the processing of human post-mortem nervous tissue to accurately phenotype T-cells in human TG ex vivo and in situ.

**Methods:**

Peripheral blood mononuclear cells (PBMC; 5 blood donors) were incubated with several commercial tissue digestion enzyme preparations to determine off-target effect on simultaneous detection of 15 specific T-cell subset markers by flow cytometry. Next, optimized enzymatic digestion was applied to ex vivo phenotype T-cells in paired PBMC, normal appearing white matter (NAWM) and TG of 8 deceased brain donors obtained < 9 h post-mortem by flow cytometry. Finally, the phenotypic and functional markers, and spatial orientation of T-cells in relation to neuronal somata, were determined in TG tissue sections of five HSV-1-latently infected individuals by multiparametric in situ analysis.

**Results:**

Collagenase IV digestion of human nervous tissue was most optimal to obtain high numbers of viable T-cells without disrupting marker surface expression. Compared to blood, majority T-cells in paired NAWM and TG were effector memory T-cells expressing the canonical T_RM_ markers CD69, CXCR6 and the immune checkpoint marker PD1, and about half co-expressed CD103. A trend of relatively higher T_RM_ frequencies were detected in TG of latently HSV-1-infected compared to HSV-1 naïve individuals. Subsequent in situ analysis of latently HSV-1-infected TG showed the presence of cytotoxic T-cells (TIA-1^+^), which occasionally showed features of proliferation (KI-67^+^) and activation (CD137^+^), but without signs of degranulation (CD107a^+^) nor damage (TUNEL^+^) of TG cells. Whereas majority T-cells expressed PD-1, traits of T-cell senescence (p16INK4a^+^) were not detected.

**Conclusions:**

The human TG represents an immunocompetent environment in which both CD4 and CD8 T_RM_ are established and retained. Based on our study insights, we advocate for T_RM_-targeted vaccine strategies to bolster local HSV-1-specific T-cell immunity, not only at the site of recurrent infection but also at the site of HSV-1 latency.

**Supplementary Information:**

The online version contains supplementary material available at 10.1186/s12974-022-02611-x.

## Introduction

T-cells protect the body from invading pathogens. Upon infection, naive T-cells (T_NA_) are activated and differentiate into effector T-cells in secondary lymphoid organs. When infection is cleared memory T-cells subsets, central memory (T_CM_) and effector memory T-cells (T_EM_) are formed [1]. About two decades ago, a new memory T-cell subset was discovered in tissues which are in frequent contact with pathogens, including the lung, gut and skin [2–6]. These memory T-cells are coined tissue-resident memory T-cells (T_RM_) that in most murine and human tissues co-express specific surface markers including CD69 (C-type lectin), αE(CD103)β7 (E-cadherin receptor), chemokine receptor CXCR6 and the inhibitory receptor programmed death receptor 1 (PD1) associated with T-cell exhaustion [6, 7]. Recently, T_RM_ have also been detected in the human brain, which has long been considered as an immune-privileged site [8]. The role of these T-cells in the human brain is still unclear, varying from a beneficial role to protect the central nervous system (CNS) from infections to their potential deleterious role in neurodegenerative diseases including multiple sclerosis [7, 8].

Viral infections of the CNS are rare though an important cause of morbidity and mortality worldwide [10]. Two human neurotropic alpha-herpesviruses, varicella-zoster virus (VZV) and herpes simplex virus-1 (HSV-1), infect and establish a lifelong latent infection in neurons of sensory and autonomic ganglia [10]. Latently infected individuals are at risk of intermittent reactivation of latent virus causing recurrent infections [11–13]. Whereas most adults worldwide are latently infected with VZV, seroprevalence of HSV-1 infection ranges between 50% to more than 90% [13]. HSV-1 and VZV infections are controlled by both innate and adaptive immunity [7]. In contrast to VZV, HSV-1 latency is considered to be maintained by local virus-specific CD4 and particularly CD8 T-cells that occasionally form clusters around latently HSV-1-infected neurons in trigeminal ganglia (TG), the main site of latency of both viruses in humans [14–17]. The active role of ganglion-resident CD8 T-cells to prevent HSV-1 reactivation has been elegantly shown in experimental HSV-1 mouse models, involving noncytolytic mechanisms mediated by interferon γ (IFN γ) and granzyme B (grB) [16, 17]. Analogous to mice, human TG are infiltrated by T-cells of a late memory phenotype that express the activation marker CD137, and the cytotoxic T-cell markers TIA-1 and grB [18–20]. Whereas T-cells in latently HSV-1-infected mouse and human ganglia express CD69, besides the canonical T_RM_ marker also expressed by T-cells early upon activation, their potential T_RM_ phenotype has not yet been demonstrated [14, 21, 22].

To investigate tissue-infiltrating T-cells, various collagenases and proteases in combination with mechanical trituration have been used by us and other groups to prepare single-cell suspensions of the tissue of interest [8, 20, 23–27]. Solely mechanical trituration is not preferred to release cells deeply infiltrated within tissues, a common feature of T_RM_ [6, 7]. Because collagens are the major protein component of extracellular matrix, collagenases are preferentially used in preparing cells for ex vivo phenotyping and in vitro cell culture [4, 23, 25]. However, variation in extracellular matrix composition between tissues and the cell type of interest demand careful selection of commercial enzyme preparations, which besides their specific protein cleavage pattern also often contain contaminations of other enzymes with unexpected protein degradation properties, to release the respective cells unharmed from their surroundings. Besides obtaining high numbers of viable single cells from tissue, preservation of surface markers is warranted to accurately phenotype the respective cell type ex vivo.

The aim of this study was twofold. First, to develop an optimized digestion protocol for human nervous tissue that fulfills the aforementioned demands by testing several different collagenases and proteases back-to-back on blood, and subsequently normal appearing white matter (NAWM) from deceased human brain donors. Second, to determine the phenotype and spatial orientation of T-cells in relation to neuronal somata in latently HSV-1-infected human TG, with special emphasis on their potential T_RM_ phenotype.

## Materials and methods

### Study participants

Human peripheral blood was obtained from healthy donors upon informed consent in accordance with local ethical committee approval (Sanquin Research, Amsterdam, The Netherlands), the Declaration of Helsinki and the Dutch rules with respect to the use of human materials from volunteer donors. Paired nervous tissue biopsies and peripheral blood were collected from deceased brain donors by the Netherlands Brain Bank (NBB; Amsterdam, The Netherlands). The NBB obtained in advance written informed consent for brain autopsy, use of clinical specimens and clinical information for research purposes from all study participants. All procedures of the NBB have been approved by Ethics Committee of Amsterdam University Medical Center (VUmc, Amsterdam, The Netherlands; project number 2009/148) and in accordance with the Declaration of Helsinki. For the present study, eligibility criteria were: acquisition of paired peripheral blood, both left and right TG specimens and a macroscopically defined NAWM biopsy (4 × 4 cm^2^) with a post-mortem interval < 10 h. Information of study participants is presented in Table 1.

**Table 1:** General characteristics of brain donors

Donor ID^*^	Underlying neurological disease	Gender	Age (years)	Cause of death	CSF (pH)	PMI (h)	VZV serostatus	HSV-1 serostatus
20-031 (○)	Alzheimer’s disease	Male	72	Terminal delirium	6.4	4:30	Positive	Negative
20-035 (□)	Non-demented control	Male	89	Euthanasia	6.7	3:05	Positive	Negative
20-099 (□)	Obsessive compulsive disorder	Female	57	Pancreatic cancer	6.3	9:30	Positive	Negative
20-112	Vascular dementia	Female	101	Pneumonia and dementia	6.5	5:25	Positive	Positive
20-118	Parkinson’s disease	Male	74	Aspiration pneumonia	6.1	8:50	Positive	Negative
21-001 (◊)	Alzheimer’s disease	Female	85	Dehydration with pneumonia	5.9	6:15	Positive	Positive
21-002	Multiple sclerosis	Female	59	Euthanasia	6.7	7:30	Positive	Positive
21-093	Autism	Male	83	Suicide	5.3	8:55	Positive	Positive
21-105	Schizophrenia	Female	90	Old age	6.8	9:45	Positive	Positive

CSF, cerebrospinal fluid; PMI, post-mortem interval

**Fig. 1:**
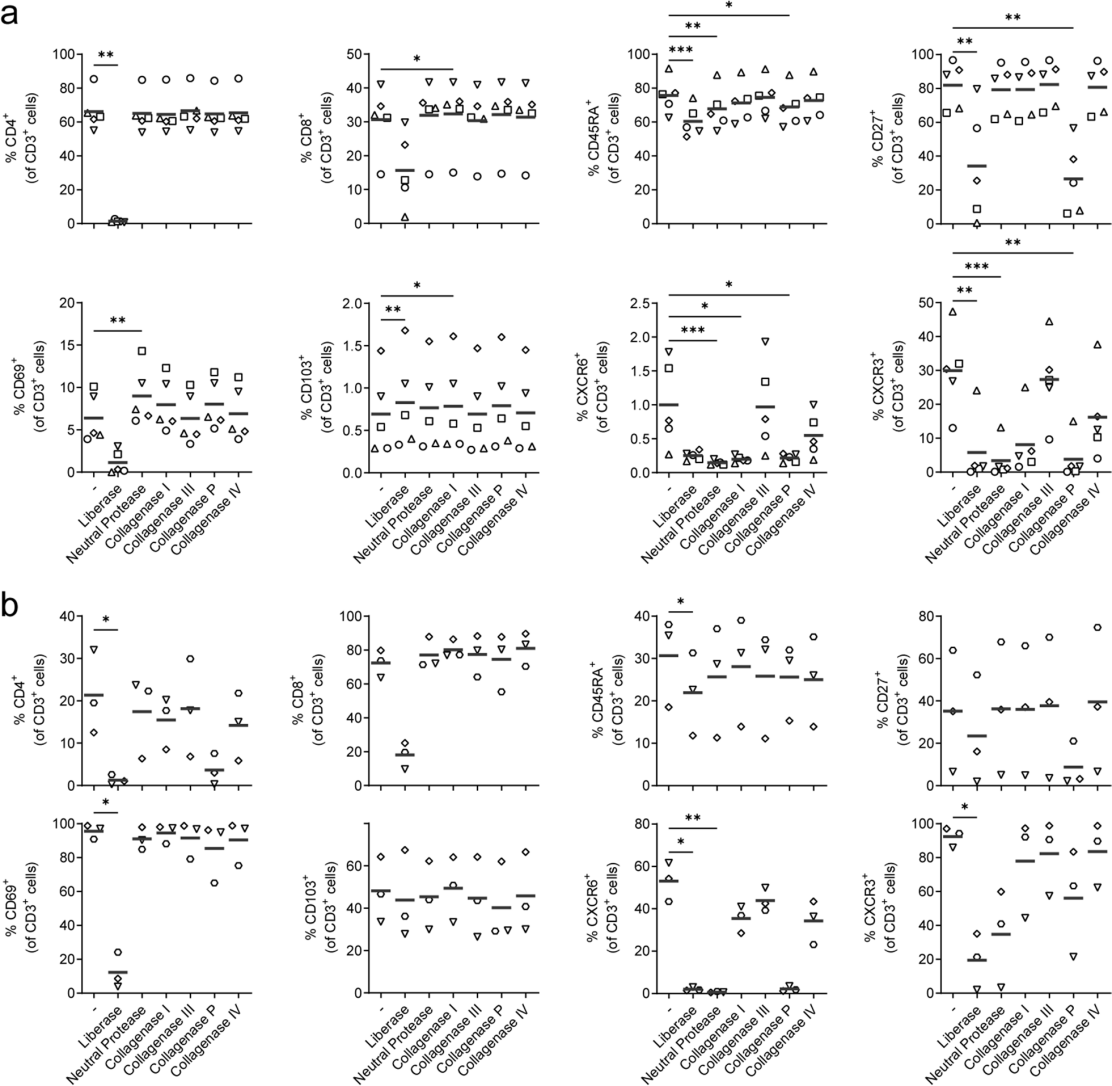
Tissue digestion enzymes show off-target effect on the detection of specific markers by flow cytometry on T-cells recovered from human peripheral blood and human brain tissue. Mononuclear cells were isolated from healthy blood donors (**a**; *n* = 5) and normal appearing white matter of deceased brain donors (**b**; *n* = 3). Frequencies of CD4^+^, CD8^+^, CD45RA^+^, CD27^+^, CD69^+^, CD103^+^, CXCR6^+^ and CXCR3^+^ T-cells (CD3^+^ cells) were quantified by flow cytometry. Bars show mean value. Each dot represents data obtained from one individual (see Table 1 for reference to brain donors in panel 'b’). All groups were compared with the group not treated with enzymes (−) and *p* values were calculated using Friedman test with Dunn’s multiple comparisons test. * *p* < 0.05; ** *p* < 0.001 and *** *p* < 0.0001

**Fig. 2:**
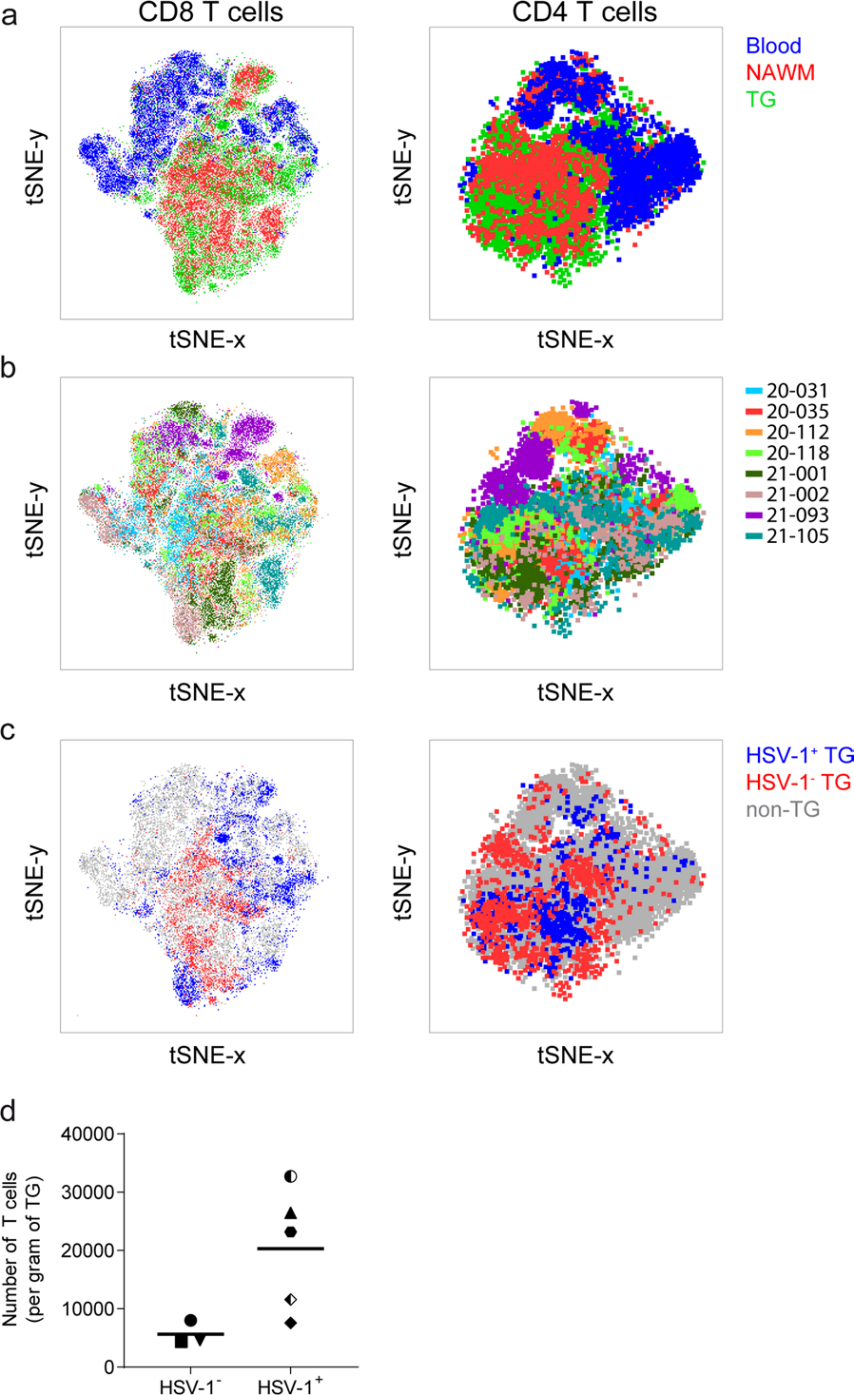
CD8 and CD4 T-cells recovered from human nervous tissue show tissue-specific and inter-individual differences in marker expressions. Mononuclear cells were isolated from paired peripheral blood (blood), normal appearing white matter (NAWM) and trigeminal ganglia (TG) of deceased brain donors (*n* = 8). **a**–**c** tSNE plot of combined flow cytometric data on paired blood-, NAWM- and TG-derived CD8 (left panel) and CD4 (right panel) T-cells. Data are grouped in colors to indicate origin specimen (**a**), origin donor (**b**), and HSV-1 infection status of TG donor: HSV-1 naïve (HSV-1^−^) and latently HSV-1-infected donors (HSV-1^+^) (**c**). **d** Absolute number of CD3^+^ T cells normalized to gram of TG in HSV-1^−^ and HSV-1^+^ donors

**Fig. 3:**
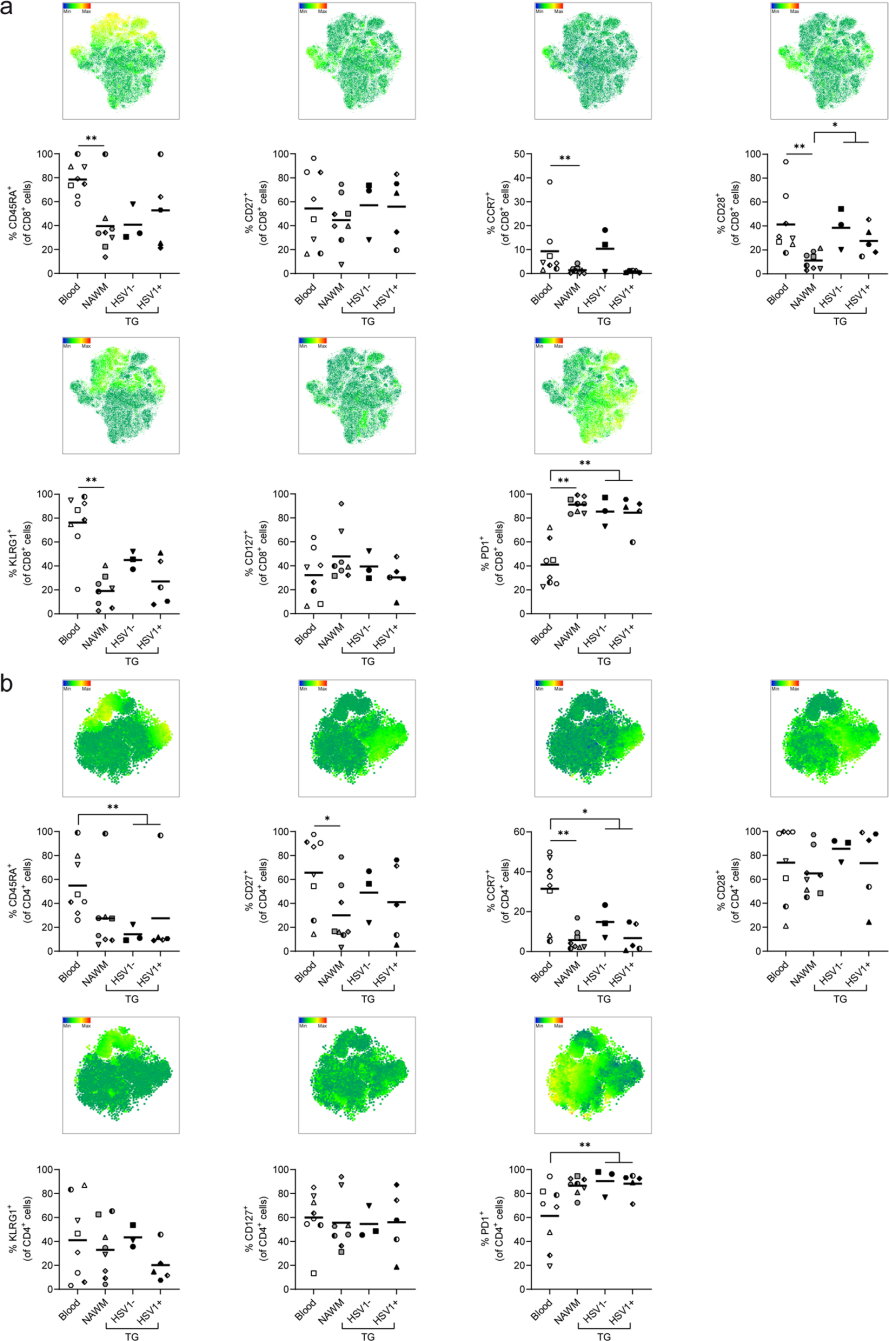
CD4 and CD8 T-cells recovered from human brain and trigeminal ganglia express an effector memory phenotype. Mononuclear cells were isolated from paired peripheral blood (blood), normal appearing white matter (NAWM) and trigeminal ganglia (TG) of deceased brain donors (*n* = 8). Frequencies of CD45RA^+^, CD27^+^, CCR7^+^, CD28^+^, KLRG1^+^, CD127^+^ and PD1^+^ CD8 (**a**) and CD4 (**b**) T-cells were quantified using flow cytometry. Each dot represents data obtained from one individual; see Table 1 for reference symbols used for each brain donors, and bars show mean value. P values were calculated using Friedman test with Dunn’s multiple comparisons test between blood, NAWM and TG (not discriminating between HSV1^−^ and HSV1^+^ TG). Mann–Whitney test was performed to compare TG-derived CD8 and CD4 T-cells recovered from HSV-1 naïve (HSV-1^−^) with latently HSV-1-infected individuals (HSV-1^+^). * *p* < 0.05 and ** *p* < 0.001

*Flow cytometry data obtained on specimens of the respective donors are shown with specific symbols in Figs. 1b, 2, 3, 5. Data of donor 20-099 are solely shown in Additional file 2: Fig. S2 and Additional file 3: Fig. S3

### Isolation of mononuclear cells from peripheral blood, TG and NAWM tissue

Peripheral blood mononuclear cells (PBMCs) were isolated from heparinized peripheral blood samples with standard density gradient technique using Ficoll-Paque (GE Healthcare Life Sciences) and were used fresh in subsequent experiments. Paired TG and NAWM tissue were stored at 4 °C in Hibernate-A medium (Invitrogen) until workup, principally within 2–15 h after dissecting the tissues by the NBB. Mononuclear cells were isolated from nervous tissues as previously described [28]. In short, the tissue was weighed, cut into small pieces and incubated for 15 min at 37 °C in digestion medium consisting of IMDM with 10% heat-inactivated fetal bovine serum (FBS), sodium pyruvate (ThermoFisher), non-essential amino acids (ThermoFisher), 1 mg/ml trypsin inhibitor (ThermoFisher) and 100 µg/ml DNAse type I (Worthington). Different types of enzymes were added at specific concentrations: Liberase (200 µg/ml, Research grade, Roche), neutral protease (1 U/ml; Nordmark Biochemicals), collagenase type I (1 mg/ml; Worthington), collagenase type III (1 mg/ml; Worthington), collagenase type IV (1 mg/ml; Worthington) or collagenase P (1 mg/ml; Worthington). Subsequently, tissues and/or PBMC were incubated for 1 h or other incubation times at 37 °C in digestion medium with or without enzyme while shaking. Before, half-way and after the digestion, the tissue was mechanically dissociated using the gentleMACS Tissue Dissociator (Miltenyi). A single-cell suspension was generated using a 100-µm cell strainer (Corning) and cells were incubated with 50 µg/ml DNAse type I for 15 min at 37 °C. To remove debris and myelin and isolate mononuclear cells, cells were resuspended into 44% Percoll (GE Healthcare Life Sciences), layered on top of 66% Percoll and centrifuged at 800 g for 20 min at room temperature. The top layer and pellet were discarded and cells were washed twice with PBS/0.1% bovine serum albumin (BSA; Sigma-Aldrich).

### Flow cytometry

Mononuclear cells, recovered from blood and nervous tissue, were washed with PBS supplemented with 0.5% BSA and 3 mg/ml human globulin G (Sanquin Reagents). Cells were incubated simultaneously for 30 min at 4 °C with specific fluorochrome-conjugated monoclonal antibodies (mAbs) directed to the following cell surface markers: CD3, CD4, CD8, CD27, CD28, CD45, CD45RA, CD69, CD103, CD127, CXCR3, CXCR6, CCR7, KLRG1 and PD1. The anti-TCRγδ mAb and Near-IR fixable dye (Invitrogen) were used to exclude γδ T-cells and dead cells from the analysis, respectively. Cells were washed twice with PBS containing 0.1% BSA and samples were measured with a FACSymphony (BD). Absolute cell counts in TG were obtained by including 50.000 CountBright™ Absolute Counting Beads (Invitrogen) to the samples just before measuring. The analysis was performed using FlowJo Version 10 software (Treestar). Gating strategy is depicted in Additional file 1: Fig. S1 and the details of antibodies used in this manuscript are presented in Additional file 6: Table S1.

### Multimarker analysis using t-distributed stochastic neighborhood embedding

Live CD4 or CD8 T-cells were gated in Flowjo v10.6.1 analysis software and exported as separate fcs-files for eight different patients. CD4 and CD8 T-cells from the different tissue compartments of all patients were randomly downsampled to 138 and 724 events, respectively, and subsequently concatenated into 1104 and 5792 events, respectively, using plugins in Flowjo v10.6.1 to normalize contribution between patients. Next, concatenated samples were analyzed using the t-distributed stochastic neighborhood embedding (tSNE) plugin in Flowjo v10.6.1. tSNE is a technique in which high-dimensional data are reduced in a non-linear manner into a low-dimensional map that preserves distances between pairs of points [29]. Basically, it performs dimensionality reduction, allowing visualization of complex multi-dimensional data in fewer dimensions while still maintaining the structure of the data” (Flowjo v10 Documentation). The tSNE analysis was performed using the standard settings. CCR7, CD103, CD127, CD27, CD28, CD45RA, CD69, CXCR3, CXCR6, KLRG1 and PD1 were selected as tSNE parameters.

### In situ analysis

Human TG cryosections (8 µm), sectioned from TG specimens of latently HSV-1-infected individuals (*n* = 5), were fixed with acetone containing 0.05% H_2_O_2_ for 10 min at room temperature. Next, serial consecutive TG sections were incubated with 5% normal goat serum for 30 min followed by overnight incubation at 4 °C with seven specific combinations of 3 unconjugated primary antibodies simultaneously, diluted in PBS with 0.1% BSA. The markers and combination thereof were designed to detail the phenotype, function and status of tissue-infiltrating T-cells in situ (see Additional file 6: Table S1). Subsequently, sections were incubated for 1 h with fluorescently labeled secondary polyclonal antibodies diluted in PBS supplemented with 0.1% BSA and 1% normal human pooled serum. To detect apoptotic cells, TUNEL staining was performed on sections using the Apoptag S7111 kit (Millipore) according to the manufacturer’s instructions. Briefly, sections were treated with 3% H_2_O_2_ and 1% methanol for 30 min, blocked with Tris-buffered saline (TBS) containing 0.1% BSA and incubated overnight at 4 °C with mouse anti-human Ki-67 diluted in TBS supplemented with 0.1% BSA and 0.3% Triton X-100 (TBS-TX). Sections were subsequently incubated for 1 h with polyclonal goat anti-mouse IgG1 labeled with Alexa Fluor 647 diluted in TBS-TX.

For all stainings, nuclei were stained with Hoechst 33342 for 10 min and subsequently mounted in Prolong Diamond Antifade Mountant (ThermoFisher). Fluorescent images were acquired on a Zeiss LSM700 confocal laser scanning microscope (Zeiss), and pictures analyzed with ZEN 2010 software (Zeiss) solely to adjust brightness and contrast.

### Statistical analysis

**Fig. 4:**
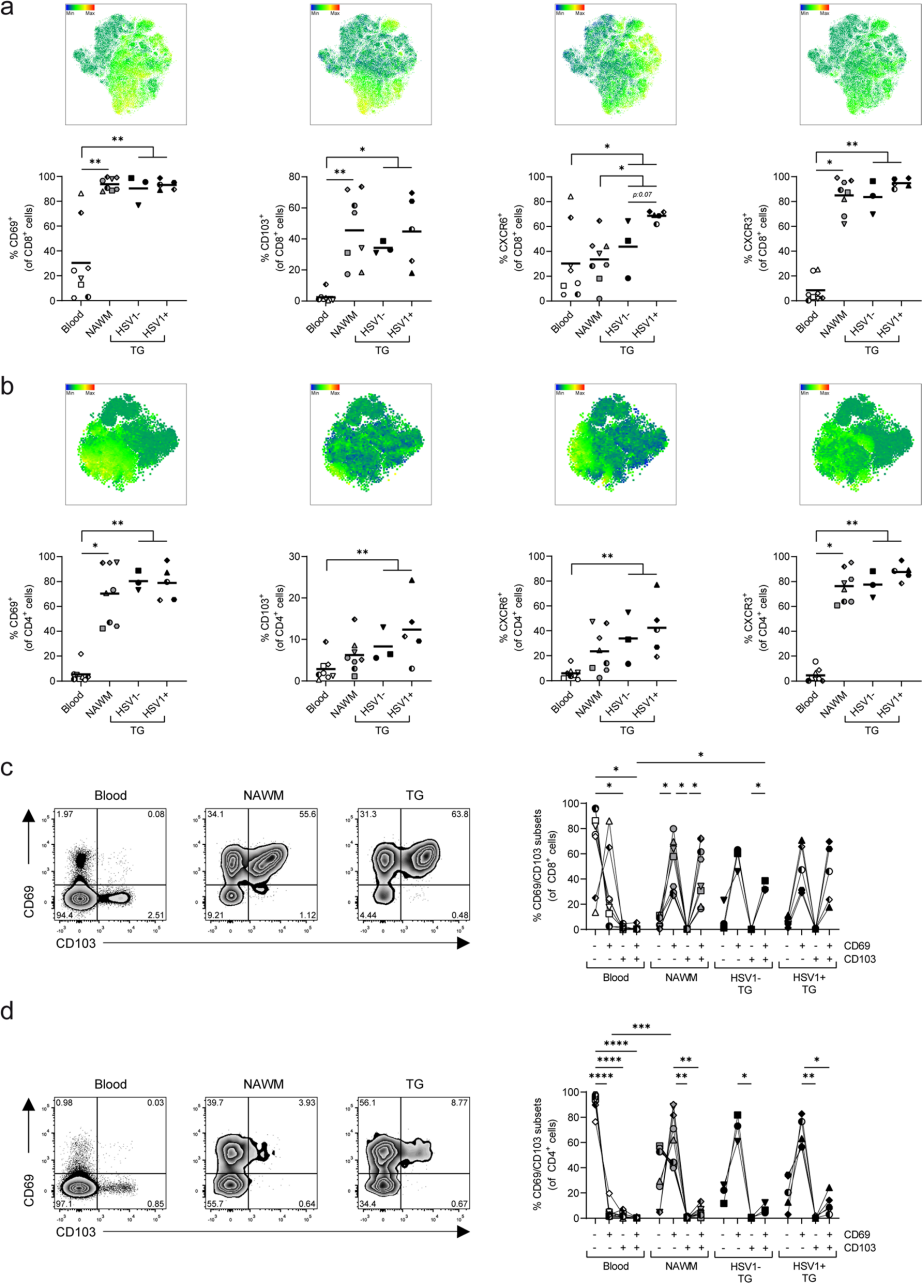
CD4 and CD8 T-cells recovered from human brain and trigeminal ganglia express a T_RM_ phenotype. Mononuclear cells were isolated from paired peripheral blood (blood), normal appearing white matter (NAWM) and trigeminal ganglia (TG) of deceased brain donors (n = 8). Frequencies of CD69^+^, CD103^+^, CXCR6^+^ and CXCR3^+^ CD8 (**a**) and CD4 (**b**) T-cells were quantified by flow cytometry. Representative dot plots (left panels) and quantification (right panels) of CD69 and CD103 co-expression on CD8 (**c**) and CD4 (**d**) T-cells. Each dot represents data obtained from one individual; see Table 1 for reference symbols used for each brain donors, and bars show mean value. P values were calculated using Friedman test with Dunn’s multiple comparisons test between blood, NAWM and TG (not discriminating between HSV1^−^ and HSV1^+^ TG). Mann–Whitney test was performed to compare TG-derived CD8 and CD4 T-cells recovered from HSV-1 naïve (HSV-1^−^) with latently HSV-1-infected individuals (HSV-1^+^). * *p* < 0.05; ** *p* < 0.001 and *** *p* < 0.0001. In **c**, **d**, all CD69/CD103 subsets from blood, NAWM, HSV1^−^ and HSV1^+^ TG are compared with each other using two-way ANOVA with Tukey’s multiple comparisons test

Statistical analysis was performed using GraphPad Prism (version 9; Graphpad Software). Data were analyzed with Friedman’s test one-way ANOVA (Figs. 1, 3, 4, Additional file 2: Fig. S2 and Additional file 3: Fig. S3), two-way ANOVA with Tukey’s multiple comparisons test comparing CD69/CD103 subsets in blood, NAWM, HSV1^−^ TG and HSV1^+^ TG (Fig. 4c and d) and Mann–Whitney test to compare HSV1^−^ vs HSV1^+^ TG (Figs. 3, 4 and Additional file 4: Fig. S4). Friedman’s test one-way ANOVA was used in Figs. 3 and 4 to compare blood, NAWM and TG (not discriminating between HSV1^−^ and HSV1^+^ TG) and the Mann–Whitney test used to compare HSV1^−^ vs HSV1^+^ TG (Figs. 3, 4). Results were considered significant at *p* < 0.05. Significance was depicted as * (*p* < 0.05) or ** (*p* < 0.01)]. All other statistical differences between groups were not significant.

## Results

### Commercial tissue digestion enzyme preparations show off-target effects that prevent reliable detection of specific T-cell subset markers by flow cytometry

We tested six different commercially available enzyme preparations back-to-back, including those used by us and others previously to digest human tissues such as lung, brain and TG [8, 20, 23–27], for their potential off-target digestion of 15 surface proteins commonly used to determine the subtype, activation and differentiation status of human T-cells ex vivo [1, 30, 31]. The expression profiles of surface proteins to differentiate human T-cells into T_NA_, T_CM_, T_EM_, T_EMRA_, and T_RM_ subsets are depicted in Additional file 7: Table S2. Frequencies of these T-cell subsets in control PBMC of healthy adults were in accordance with prior studies (Fig. 1a and Additional file 2: Fig. S2a, b) [8, 28, 32]. However, neutral protease, collagenase type P and particularly liberase treatment of PBMC impaired detection of the majority of the T-cell subset-defining markers significantly (Fig. 1a). Detection of the chemokine receptor CCR7, CD28, interleukin 7 receptor alpha (IL-7Rα; CD127) and the T-cell terminal differentiation marker (KLRG1) was not impaired (Additional file 2: Fig. S2a, b). In conclusion, the data demonstrate that several commercial tissue digestion enzyme preparations show unwanted off-target effects on the epitopes recognized by the mAbs used.

### Collagenase type I, III and IV facilitate correct detection of T-cell subset markers by flow cytometry on T-cells recovered from human nervous tissue

To determine whether these off-target effects also occur within the context of the tissue architecture, we used the same enzymes to phenotype T-cells recovered from cadaveric human brain tissue. NAWM was selected to compare our flow cytometry data with earlier studies by others [8, 33]. The NAWM specimens were obtained < 10 h post-mortem from individuals who suffered from various neurodegenerative diseases (*n* = 3; Table 1). Neutral protease, collagenase type P and particularly liberase impaired detection of the same markers on cadaveric NAWM- as blood-derived T-cells (Fig. 1b and Additional file 2: Fig. S2c). Compared to T-cells obtained by solely mechanical trituration of NAWM tissue, which only very inefficiently releases cells like T_RM_ that are deeply infiltrated within tissues [6, 7], collagenase type I, III and IV digestion of NAWM did not significantly change detection of the T-cell markers tested. Next, we determined the collagenase type IV’s optimal digestion time (i.e., 30, 60 or 120 min) and ratio of NAWM weight and digestion medium volume (i.e., 1:2.5, 1:5 or 1:10 weight/volume ratio). Whereas the weight/volume ratio did not alter marker detection, CXCR6 detection was inversely correlated with duration of collagenase type IV digestion of NAWM (Additional file 3: Fig. S3).

In conclusion, the data demonstrate that the choice of tissue digestion enzyme has a major effect on the detection of specific cell surface markers commonly used to phenotype T-cells in human tissues by ex vivo flow cytometry [1, 30, 31]. The data demonstrate that collagenase type IV (1 mg/ml) treatment of human nervous tissue biopsies for 60 min at 37 °C is most optimal to characterize T-cell subsets from human nervous tissue specimens.

### Human TG-derived T-cells express canonical T_RM_ markers

We analyzed the phenotype of human TG-infiltrating T-cells and compared it with T-cells recovered from blood and NAWM samples of the same individual by multiparametric flow cytometry (*n* = 8 brain donors; Table 1). tSNE analysis method was performed on this high-dimensional flow cytometry within CD4 and CD8 T-cells. Unsupervised clustering on CCR7, CD103, CD127, CD27, CD28, CD45RA, CD69, CXCR3, CXCR6, KLRG1 and PD1 expression showed that blood CD4 and CD8 T-cells segregate from their counterparts recovered from NAWM and TG (Fig. 2a). tSNE clusters were mainly formed by inter-individual differences in marker expression (Fig. 2b). Concurrent with earlier studies [8, 20], both NAWM and TG contained more CD8 than CD4 T-cells and the CD4/CD8 ratio was similar between both nervous tissue compartments (Figs. 2 and 3). A high proportion of NAWM- and TG-derived CD4 and CD8 T-cells did not express CD45RA and CCR7, indicating low numbers of T_NA_ and T_CM_ cells in these tissues (Fig. 3). NAWM, but not TG, contained significantly lower frequencies of CD28^+^ CD8 T-cells (Fig. 3a) and CD27^+^ CD4 T-cells compared to paired PB (Fig. 3b). However, CD27 and CD28 expression were lower in both NAWM- and TG-derived T-cells implicating their T_EM_ phenotype (data not shown). Frequencies of CD8 and CD4 T-cells expressing the inhibitory marker programmed cell death protein 1 (PD1) were increased in NAWM and TG. Finally, the proportion of KLRG1^+^ and CD127^+^ CD8 and CD4 T-cells remained unaltered or decreased between blood and both nervous tissues (Fig. 3).

The segregation of NAWM- and TG-derived T-cells compared to paired blood-derived T-cells in the tSNE analysis was due to increased frequencies of CD69^+^, CD103^+^, CXCR6^+^ and CXCR3^+^ cells of both NAWM- and TG-derived CD8 and CD4 T-cells compared to their blood counterparts (Fig. 4a, b). About 40–50% of CD8 T-cells expressed CD103 (Fig. 4a), and co-expression analysis showed that CD69^+^CD103^−^ and CD69^+^CD103^+^ cells were the dominant CD8 T-cell subsets in both human NAWM and TG (Fig. 4c). In contrast to CD8 T-cells, the majority of NAWM- and TG-derived CD4 T-cells did not express CD103, with TG-derived CD4 T-cells having the highest frequency of CD103^+^ cells (Fig. 4b). Indeed, CD69^+^CD103^−^ cells were the dominant CD4 T-cell population in both NAWM and TG (Fig. 4d).

Finally, we related the T_RM_ phenotypes to the individuals’ HSV-1 and VZV infection status. Whereas all individuals were VZV infected, 5 of 8 subjects were latently HSV-1 infected (Table 1). Unsupervised clustering revealed that CD8 and CD4 T-cells recovered from TG of latently HSV-1-infected individuals (HSV-1^+^) did not cluster with T-cells from HSV-1 naïve individuals (HSV-1^−^), suggesting differences in T-cell phenotype (Fig. 2c). Notably, a trend of higher T-cell numbers per gram TG was detected in HSV-1^+^ compared to HSV-1^−^ individuals which consistent with the role of T-cells controlling HSV-1 latency in human TG analogous to the HSV-1 mouse model (Fig. 2d) [9, 17]. TG of HSV-1^+^ individuals showed a tendency for lower frequencies of CD8 and CD4 T-cells expressing T_CM_ markers (e.g., CCR7 and CD28; Fig. 3), and concomitant higher frequencies of CD8 and CD4 T-cells expressing T_RM_ markers (e.g., CD103, CD69 and CXCR6) (Fig. 4). Frequency and expression levels of these markers were detailed within CD69/CD103 subsets between HSV-1- TGs and HSV-1^+^ TGs (Additional file 5: Fig. S5). This analysis revealed clear trends and some significant increases in PD1 and CXCR3 expression in CD69/CD103 T-cell subsets, but due to availability of low number HSV-1^−^ TG lack sufficient statistical power. Overall, the data demonstrate that human TG- and NAWM-derived T-cells show close phenotypic resemblance in the expression of the canonical T_RM_ markers CD69, CD103 and CXCR6.

### Markers of cytotoxicity and spatial orientation of T-cells in human latently HSV-1-infected trigeminal ganglia

**Fig. 5:**
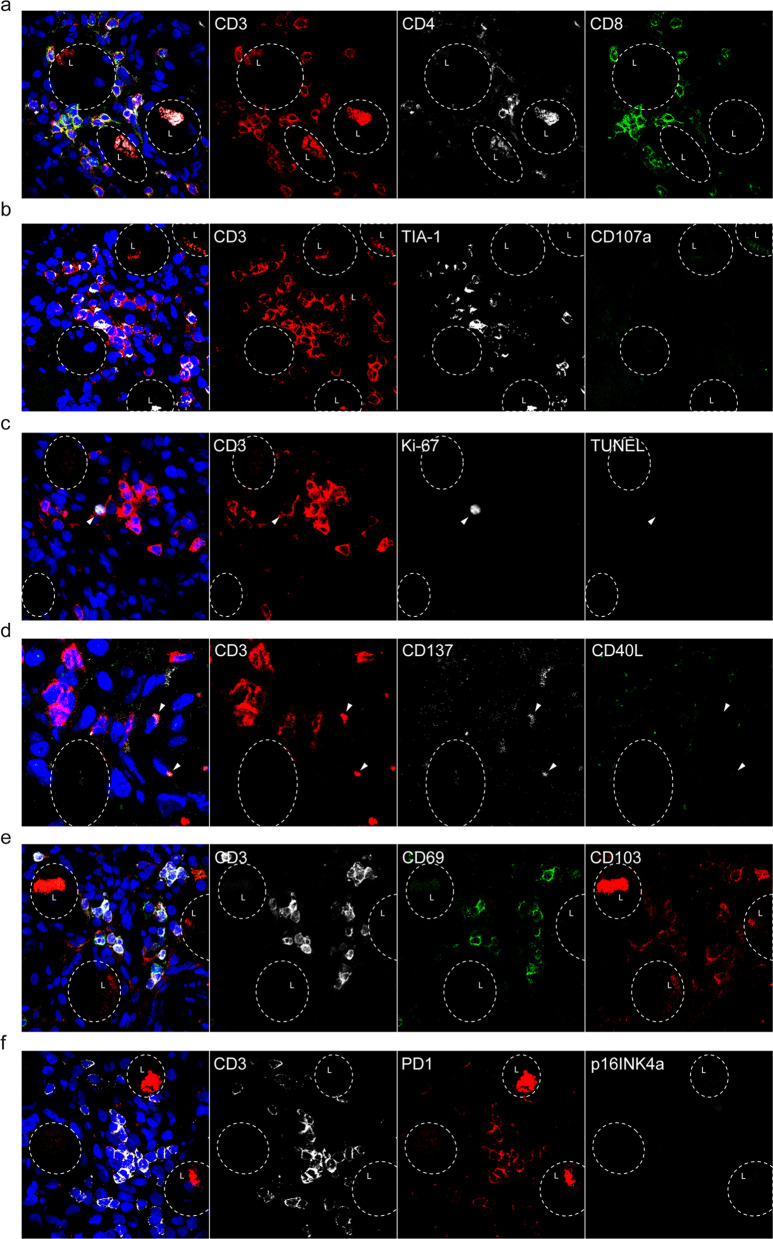
Markers of cytotoxicity and spatial orientation of T-cells in human latently HSV-1-infected trigeminal ganglia. Triple immunofluorescence staining for various markers (as indicated) in TG tissue sections (8 µm). Nuclei were stained with DAPI (blue color). Images of representative stainings are shown. **a** CD3 (red), CD4 (white) and CD8 (green) T-cells surround neurons (indicated with dotted white circles). Panel ‘a’ is zoom-in of region shown in Additional file 4: Fig. S4b. Majority of T-cells (CD3^+^ cells) express CD8. **b** CD107a (green) and TIA-1 (white) staining of T-cells (red) cells. Majority of T-cells cells express TIA-1, but not the degranulation marker CD107a. **c** TUNEL (green) and Ki-67 (white) staining of T-cells (red) cells. T-cells do not express TUNEL (late apoptosis marker), but occasionally T-cells express Ki-67 (cell proliferation marker). **d** CD137 (white) and CD40L (green) staining of T-cells (white) cells. T-cells express the activation markers CD137 and CD40L. **e** Majority of T-cells (CD3, white) express CD69 (green) of which a subset expressed CD103 (red). **f** PD1 (red) and p16INK4a (green) staining of T-cells (white) cells. Majority of T-cells express the exhaustion marker PD1, but do not express the senescence marker p16INK4a. Selected number of neuronal somata, with or without lipofuscin (L), are marked with a dotted circle. Representative images of one donor out of five are shown

To complement the flow cytometric analysis, we used intact tissue to assess markers of cytotoxic T-cells and determine the spatial orientation of T-cells in human TG, mainly T_RM_ cells based on flow cytometry, using triple immunofluorescent stainings on TG cryosections of five latently HSV-1-infected individuals. TG cryosections of HSV-1 naïve individuals (HSV-1^−^) were not analyzed as these tissues are currently not available due to rarity of HSV-1 naïve donors in our cohort (see “Discussion” section). Six marker combinations were selected to detail the phenotype, status and location of T-cells in consecutive TG sections (for marker combinations see Additional file 6: Table S1). T-cells were found throughout the TG, predominantly CD8 T-cells, which occasionally formed clusters around neuronal somata (Fig. 5a and Additional file 4: Fig. S4). Concurrent to earlier reports, T-cell clusters consisted of both CD4 and CD8 T-cells and occasionally localized adjacent to latently HSV-1-infected neurons expressing the viral latency associated transcript (LAT), although no preferential co-localization of T-cells to LAT^+^ neurons was observed [15, 18, 20]. Their cytotoxic phenotype was exemplified by expression of TIA-1, but in the absence of CD107a expression suggest that these cytotoxic T-cells did not undergo degranulation at time of tissue sampling (Fig. 5b) [34]. Indeed, no evidence of cell death, visualized by the TUNEL assay, was observed among neurons or other TG-resident cells (Fig. 5c). Local T-cell proliferation was occasionally observed by means of Ki-67 expression (Fig. 5c). While devoid of CD40L, a marker expressed by activated CD4 T-cells [35], expression of the T-cell activation marker CD137 expressed upon antigen encounter [36] was occasionally detected suggesting that these T-cells have recently encountered their cognate antigen locally (Fig. 5d). Notably, majority of T-cells expressed CD69 with a subset of them co-expressing CD103 (Fig. 5e). The occasional CD137 expression combined with generalized CD69 expression by human TG-infiltrating T-cells signifies their T_RM_ phenotype and not merely the T-cells’ activation status [32]. Whereas the majority of T-cells expressed PD1, the exhaustion marker [37], TG-resident T-cells did not show signs of cellular senescence, as T-cells did not express p16INK4a (Fig. 5f).

## Discussion

Standardized enzymatic digestion protocols are warranted to appropriately dissociate tissues and free viable cells at high numbers that preserves surface markers for accurate ex vivo phenotyping of both tissue-resident and infiltrating cell types. The current study addressed this unmet need for human nervous tissues by establishing that collagenase type IV (1 mg/ml) treatment of cadaveric human nervous tissue specimens for 60 min at 37 °C is most optimal. Using this validated protocol, we describe that the majority of T-cells in human NAWM and TG resemble and are effector memory T-cells expressing canonical T_RM_ markers.

Enzymes are frequently used in tissue digestion to phenotype tissue-infiltrating immune cells ex vivo by flow cytometry. Here, we demonstrated that collagenase type IV is most optimal to generate single-cell suspensions for the characterization and study of T-cell subsets from human NAWM and TG, the tissue samples used in this study. These findings are consistent with those of earlier studies showing that collagenase type IV similarly preserves T_RM_ surface markers on T-cells isolated from human skin [23], intestine and lung samples [1]. Discrepancies in T-cell marker expression between studies can arise when using different enzymes, but may also be related to the differences of mAb clones recognizing epitopes with different susceptibility to enzymatic digestion. These data argue for standardization of methods, especially enzymes and mAbs, to appropriately phenotype cells in human tissues, including nervous tissues.

Using our optimized digestion protocol, we demonstrated the presence of T_RM_ in both the central (NAWM) and peripheral nervous system (TG) of humans. While confirming data by others on their presence in human brain tissues [8, 33], we now provide conclusive evidence that human TG-resident T-cells express the canonical T_RM_ markers CD69, CXCR6 and PD1, and about half co-expressed CD103 [6, 7]. Notably, T_RM_ in paired NAWM and TG specimens showed a similar T_RM_ phenotype, indicated by the high correlation in the frequencies of CD27^+^, CXCR3^+^, PD1^+^, KLRG1^+^, CXCR6^+^ and CD103^+^ CD8 T-cells in paired NAWM and TG (data not shown). However, in contrast to NAWM, CD4 T-cells in TG, and mainly in HSV-1^+^ individuals, showed a tendency for a higher frequency of CD103^+^ cells [8, 33]. Whether this is due to a different tissue environment and leads to functional differences remains to be determined. In addition, CD103^+^ TG T_RM_ showed a trend of higher expression of PD1, CXCR6 and CXCR3 compared to CD103^−^ TG T_RM_. This hints to a phenotypical difference between CD103^−^ and CD103^+^ T_RM_ within human TG and might explain our observation in situ that CD103^+^ are mainly found in T-cell clusters surrounding neuronal somata. Notably, the CXCR3 ligand CXCL10 is selectively expressed in human TG by cells in neuron-interacting T-cell clusters [19]. Mice with a genetic deletion of CXCL10 or CXCR3 had reduced numbers of CD8 T-cells residing in the infected cornea and innervating TG after HSV-1 infection, suggesting that T-cells are recruited to TG via the CXCR3–CXCL10 axis [38]. The involvement of the CXCR6–CXCL16 axis in T_RM_ recruitment in the context of TG remains to date unknown and requires further investigation. Part of the canonical human T_RM_ signature [30], and analogous to T-cells recovered from human brain tissue (Fig. 2) [8], TG contained relatively high frequencies of PD1^+^ T-cells. Its ligand, PD-L1, is expressed on neuron-interacting satellite glial cells in human TG [37, 39], and previous studies have shown that the frequency of HSV-1-specific CD8 T-cells expressing the inhibitory receptors PD1 and LAG3 is increased in blood of individuals with recurrent herpetic disease compared to asymptomatic individuals [40]. Interestingly, blockade of these inhibitory receptors with antibodies increased the functional activity of CD8 T-cells in TG of HSV-1 infected rabbits and leading to reduced HSV-1 viral loads and disease severity [40]. This suggests that intermittent reactivation of HSV-1 in TG, and to a lesser extent VZV, may lead to frequent or even chronic antigenic stimulation and consequently T-cell exhaustion reducing their functional activity [41]. Although TG-infiltrating T-cells had cytotoxic potential, as indicated by TIA-1 expression, no tissue damage was observed, as indicated by a lack of TUNEL staining.

A trend for increased frequencies of CD103^+^ (Fig. 4a, b) and reduced frequencies of KLRG1^+^ T-cells (Fig. 3a, b) was observed in TG of HSV-1^+^ versus HSV-1^−^ individuals. KLRG1 and CD103 are both E-cadherin receptors, which are expressed by sensory neurons, satellite glial cells and Schwann cells in sensory ganglia [42]. Mice studies have indicated that CD8 T_RM_ may develop from tissue-infiltrating KLRG1^−^ cells [43, 44], and that forced expression of KLRG1 impedes T_RM_ formation via inhibitory immunoreceptor tyrosine-based inhibitory motif (ITIM) signals [45]. These data suggest that repression of KLRG1 is important for normal T_RM_ development and might explain the increased expression of KLRG1 in latently HSV-1-infected human TG. The role of HSV-1 latency in this T_RM_ development requires further investigation, but might indicate that control of HSV-1 latency leads to formation of T_RM_ from KLRG1^−^ precursors. Maintenance of protective T_RM_ in tissues, including skin and lung, is attributed to both de novo formation of T_RM_ from circulating memory T-cells and local proliferation of T_RM_. The Ki-67 expression by T-cells in human TG support local proliferation in T_RM_ homeostasis, essentially as described previously in HSV-1 mouse models [20, 46]. These issues need to be addressed in future studies on human TG.

This study has several shortcomings. First, trends in T-cell subset differences were observed in TG of HSV-1^+^ versus HSV-1^−^ individuals, but the low number of HSV-1^−^ TG limit statistical power. Whereas acquisition of human TG with short post-mortem interval is challenging, the high age of the cohort (average age 67 years) and consequently high prevalence of HSV-1 infection (87%) seriously limits the number of HSV-1^−^ TG available. These data warrant future studies on larger cohorts. Second, differential markers expression between dispersed single T-cells and neuron-interacting T-cell clusters is of interest but was not determined. In situ analysis on human TG for various marker combinations, including the T_RM_ markers CD69 and CD103, was performed as support of the detailed flow cytometry—but not as stand-alone data—on the phenotype and location of T cells in human TG. This technology solely provides insight into the phenotype and spatial orientation of cell types in just a thin section of a tissue. Application of in situ analysis as stand-alone technology warrants staining of very high numbers of sections covering the complete tissue of interest. A very time consuming and costly effort that does not outweigh the value of multiparametric flow cytometry of cell suspensions generated from the complete tissue. Third, ex vivo phenotyping of any cells by flow cytometry is limited by the number of markers included. The hypothesis-driven nature of this technology is a major drawback when new or large sets of markers of interest are newly described. Unbiased technologies like single-cell RNA sequencing in combination with confirmatory in situ analysis is clearly more suitable to study T_RM_ variation within and between tissues and will be used in future studies once widely available [25, 30, 42, 45].

In conclusion, we developed an optimal enzymatic digestion protocol for human nervous tissue to demonstrate that T-cells in human TG express canonical T_RM_ features, which closely resemble their counterparts in NAWM. Furthermore, in situ analysis showed that T_RM_ in TG of HSV-1-latently infected individuals show signs of local activation (CD137^+^), proliferation (Ki-67^+^), cytotoxic potential (TIA-1^+^), and most likely exhaustion (PD1^+^). This TG-residing T-cell subset is considered pivotal in providing long-term control of HSV-1 latency. Further characterization of these cells will lead to a better understanding of the generation, maintenance and function of T_RM_ in the peripheral nervous system. These insights may guide the development of new therapeutic options aimed to establish and maintain a protective T_RM_ pool in TG that protects against recurrent HSV-1 diseases.

## Supplementary Information

**Additional file 1: Figure S1.** Flow cytometry gating strategy for T-cells recovered from human peripheral blood, normal appearing white matter and trigeminal ganglia. Live leukocytes (CD45^high^) cells were selected by setting a lymphocyte gate in FSC-A and SSC-A, followed by single cell gates using FSC-W/FSC-H and SSC-W/SSC-H, a subsequent live CD45^high^ gating using the LIVE/DEAD Fixable Near-IR channel (LD) and CD45 expression. γδ T-cells were excluded as well. Next, CD3^+^CD45^+^ cells were selected and subsequent gating on CD4 and CD8 cells was performed. Frequencies of gated cells, boxed areas, are provided in each plot. Data on cells recovered from paired peripheral blood, normal appearing white matter (NAWM) and trigeminal ganglia (TG) samples of a representative deceased brain donor are shown.

**Additional file 2: Figure S2.** Liberase and neutral protease digestion greatly impair detection of markers by flow cytometry on T-cells recovered from human peripheral blood and normal appearing white matter. Mononuclear cells were isolated from unpaired peripheral blood (n = 5; a-b) and normal appearing white matter (n = 3; c). (a) Frequency of CD45^+^ and CD3^+^ cells of blood-derived lymphocytes were quantified upon treatment with different tissue digestion enzymes (as indicated). (b-c) Frequencies of CCR7^+^, CD28^+^, KLRG1^+^, CD127^+^ and PD1^+^ T-cells (CD3^+^) cells were quantified by flow cytometry. Bars show mean value. Each dot represents data obtained from one donor (see Table 1 for reference to brain donors in panel ‘c’). All groups were compared with the group without enzymes (-) and *p* values were calculated using Friedman test with Dunn’s multiple comparisons test. * *p* < 0.05; ** *p* < 0.001 and *** *p* < 0.0001.

**Additional file 3: Figure S3.** Effect of concentration and duration collagenase IV digestion of human brain tissue on the expression of T-cell differentiation markers. Normal-appearing white matter obtained from 3 deceased brain donors was digested with collagenase IV at different tissue weight to digestion medium volume ratios (i.e., 1:2.5; 1:5 or 1:10 w:v) and different digestion incubation times (i.e., 30, 60 or 120 min). Frequency of CD4^+^, CD8^+^, CD69^+^, CD103^+^, CD45RA^+^, CD27^+^, CCR7^+^, CD28^+^, KLRG1^+^, CD127^+^, PD1^+^, CXCR3^+^ and CXCR6^+^ T-cells (CD3^+^ cells) were quantified by flow cytometry in different tissue weight: digestion volume ratios (w:v) and different digestion incubation times (as indicated). Bars show mean value. Each dot represents data obtained from one individual. All groups were compared with each other and *p* values were calculated using Friedman test with Dunn’s multiple comparisons test. * *p* < 0.05.

**Additional file 4: Figure S4.** Localization of CD8 and CD4 T-cells within a latently HSV-1-infected human trigeminal ganglion. In situ analysis of a representative latently HSV-1-infected human trigeminal ganglion (TG), cut into serial 8 µm tissue sections, which was (1) conventionally stained with hematoxylin and eosin (a and c; insert shows whole TG section with rectangle region zoomed in for panels a and b, and c and d) and (2) triple immunofluorescence staining for CD3, CD4 and CD8 on a consecutive TG section (b and d). Central part of the TG containing neuronal somata, selected number of neuronal somata, with or without lipofuscin (L), are marked with a dotted circle (a and b), and peripheral region containing axon bundles and connective tissues but devoid of neuronal somata are shown (c and d). In panels b and d, T-cells (CD3^+^; red) are dispersed throughout the TG tissue, predominantly in the neuronal cell body TG region (panels a and b), where occasionally neuron-interacting T-cell clusters are found that are composed of both CD4 (white) and CD8 (green) T-cells (see squared region in panel b, which is presented in Fig. 5a). Majority of CD3^+^ cells co-expressed CD8 and nuclei were stained with DAPI (blue color). Original magnification was 5 × and scale bars illustrate size of the tissue section shown.

**Additional file 5: Figure S5.** CD69^+^CD103^+^ T-cells recovered from human trigeminal ganglia seem phenotypically different from CD69^+^CD103- T-cells. Mononuclear cells were isolated from trigeminal ganglia (TG) of deceased brain donors (n = 8). Expression levels (geometric mean fluorescence intensity, gMFI) of KLRG1, PD1, CXCR6 and CXCR3 and percentages (%) of KLRG1^+^, PD1^+^, CXCR6^+^ and CXCR3^+^ CD8 (a) and CD4 (b) T-cells were quantified within the CD69 and CD103 subsets (as indicated) by flow cytometry. Each dot represents data obtained from one individual. P values were calculated using Friedman test with Dunn’s multiple comparisons test between CD69/CD103 subsets. Mann–Whitney test was performed to compare CD69/CD103 subsets in TG-derived CD8 and CD4 T-cells between HSV-1 naïve (HSV-1^−^) and latently HSV-1-infected individuals (HSV-1^+^). * *p* < 0.05 and ** *p* < 0.001.

**Additional file 6: Table S1.** Characteristics antibodies used in this study.

**Additional file 7: Table S2.** Markers of human T-cell differentiation.

## Data Availability

The datasets used and/or analyzed during the current study are available from the corresponding author on reasonable request.

## Abbreviations

HSV-1: Herpes simplex virus type 1
mAb: Monoclonal antibody
NAWM: Normal-appearing white matter
PBMC: Peripheral blood mononuclear cells
TCL: T-cell line
TG: Trigeminal ganglion
T_RM_: Tissue-resident memory T-cell
tSNE: T-distributed stochastic neighborhood embedding
VZV: Varicella-zoster virus
