# Supplementary figures and images for: T-cells in human trigeminal ganglia express canonical tissue-resident memory T-cell markers
Source: J Neuroinflammation. 2022 Oct 6;19:249. doi: 10.1186/s12974-022-02611-x (PMC9535861; doi:10.1186/s12974-022-02611-x)

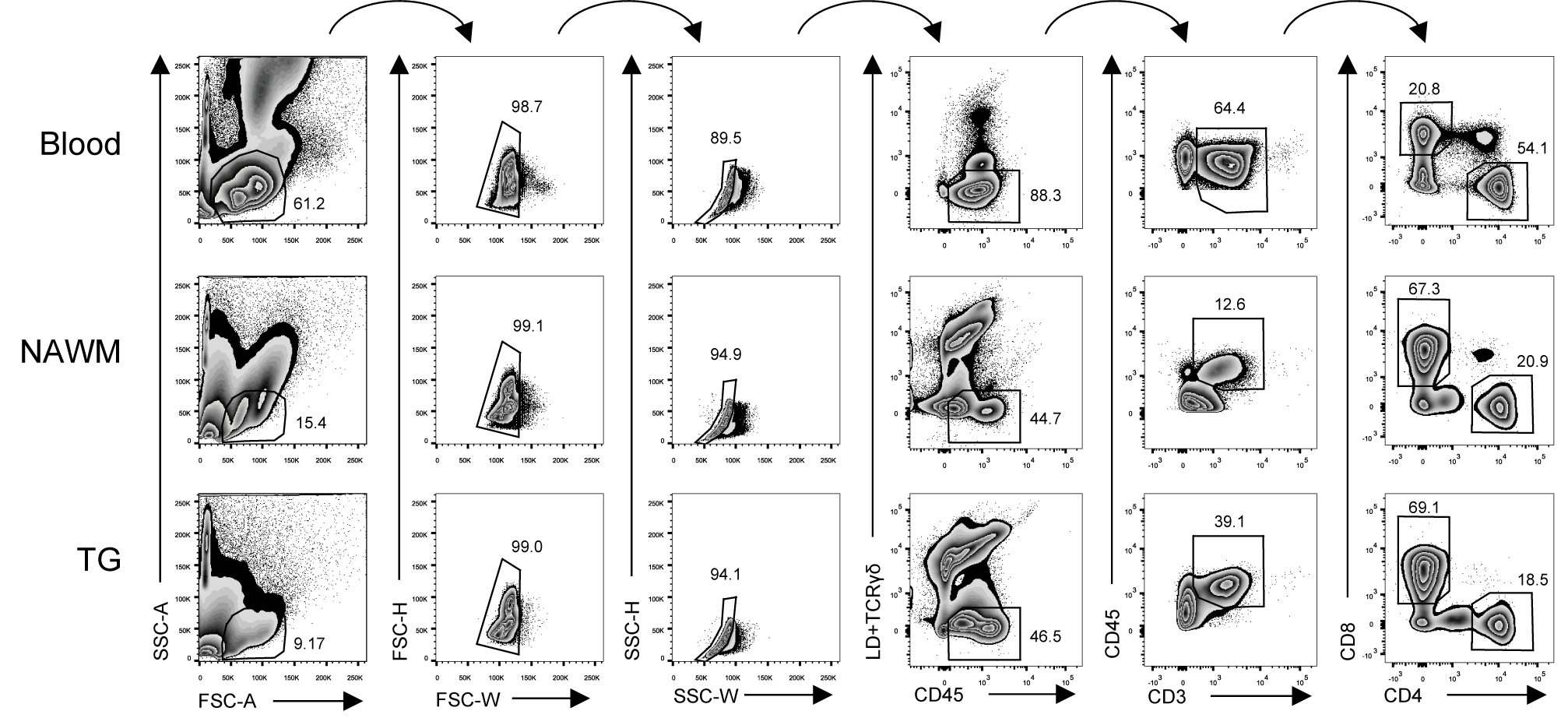

Supplement: Supplementary file 1 — Additional file 1: Figure S1. Flow cytometry gating strategy for T-cells recovered from human peripheral blood, normal appearing white matter and trigeminal ganglia. Live leukocytes (CD45high) cells were selected by setting a lymphocyte gate in FSC-A and SSC-A, followed by single cell gates using FSC-W/FSC-H and SSC-W/SSC-H, a subsequent live CD45high gating using the LIVE/DEAD Fixable Near-IR channel (LD) and CD45 expression. γδ T-cells were excluded as well. Next, CD3+CD45+ cells were selected and subsequent gating on CD4 and CD8 cells was performed. Frequencies of gated cells, boxed areas, are provided in each plot. Data on cells recovered from paired peripheral blood, normal appearing white matter (NAWM) and trigeminal ganglia (TG) samples of a representative deceased brain donor are shown. [file 12974_2022_2611_MOESM1_ESM.tif]

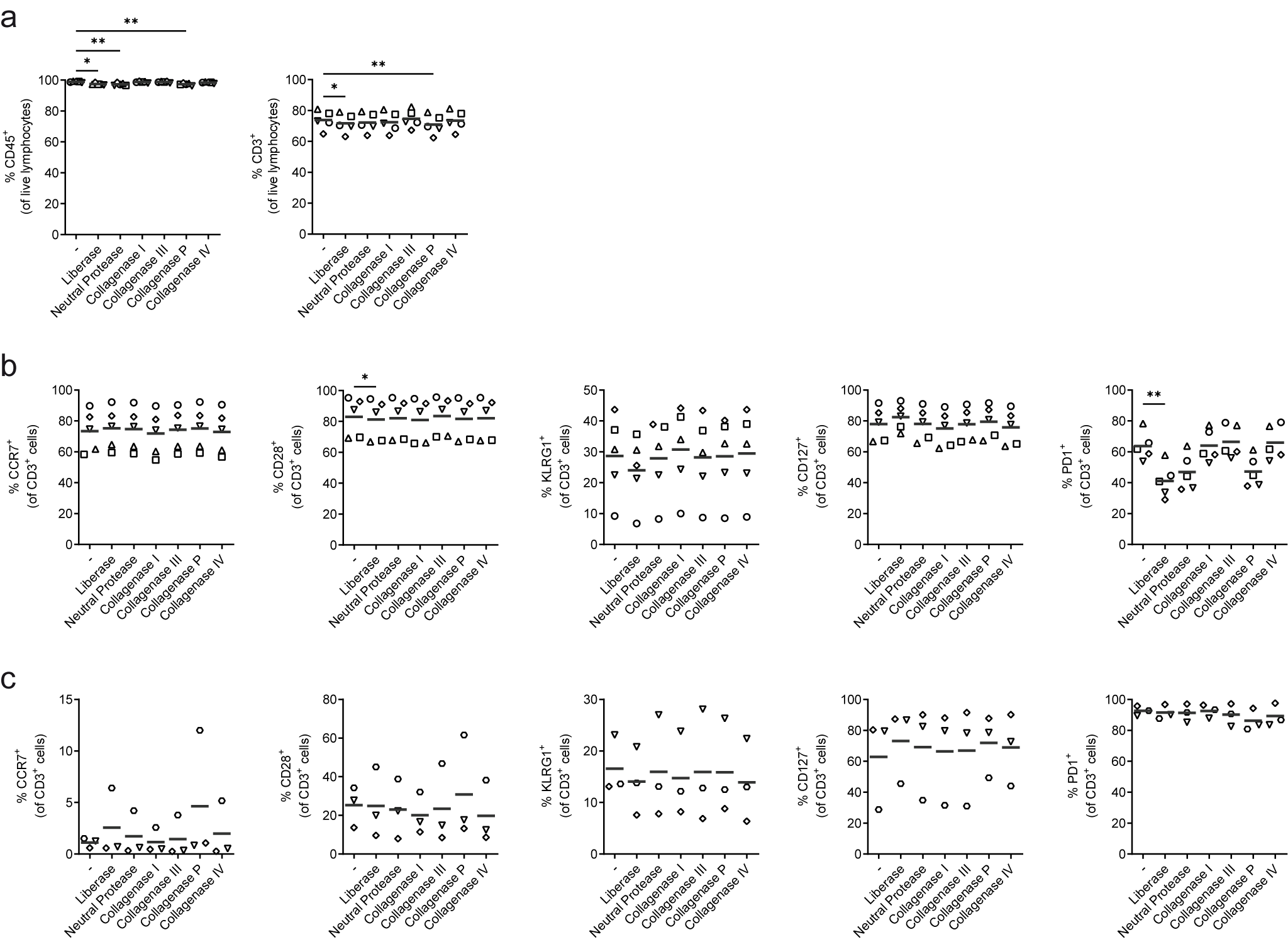

Supplement: Supplementary file 2 — Additional file 2: Figure S2. Liberase and neutral protease digestion greatly impair detection of markers by flow cytometry on T-cells recovered from human peripheral blood and normal appearing white matter. Mononuclear cells were isolated from unpaired peripheral blood (n = 5; a-b) and normal appearing white matter (n = 3; c). (a) Frequency of CD45+ and CD3+ cells of blood-derived lymphocytes were quantified upon treatment with different tissue digestion enzymes (as indicated). (b-c) Frequencies of CCR7+, CD28+, KLRG1+, CD127+ and PD1+ T-cells (CD3+) cells were quantified by flow cytometry. Bars show mean value. Each dot represents data obtained from one donor (see Table 1 for reference to brain donors in panel ‘c’). All groups were compared with the group without enzymes (-) and p values were calculated using Friedman test with Dunn’s multiple comparisons test. * p < 0.05; ** p < 0.001 and *** p < 0.0001. [file 12974_2022_2611_MOESM2_ESM.tif]

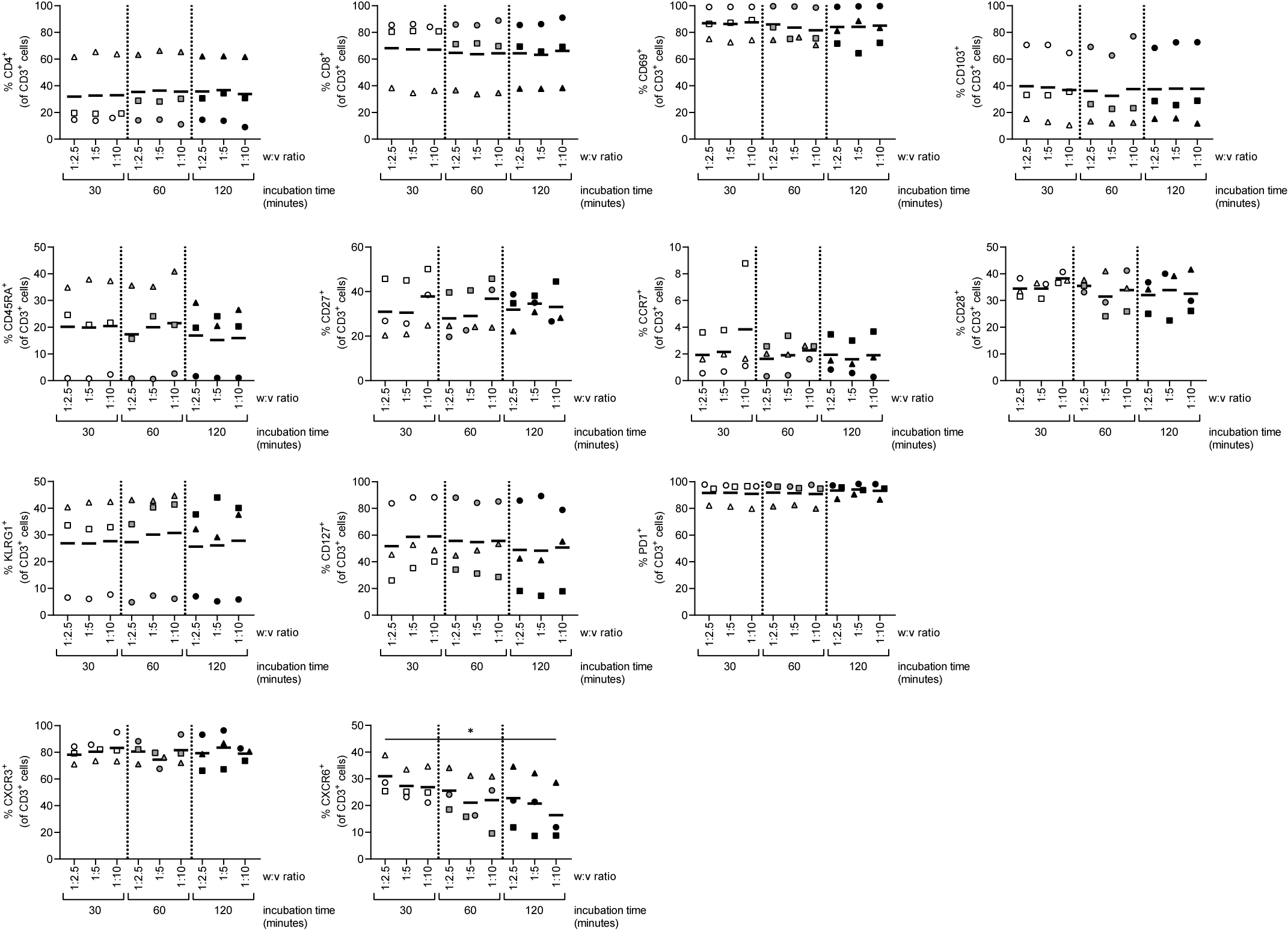

Supplement: Supplementary file 3 — Additional file 3: Figure S3. Effect of concentration and duration collagenase IV digestion of human brain tissue on the expression of T-cell differentiation markers. Normal-appearing white matter obtained from 3 deceased brain donors was digested with collagenase IV at different tissue weight to digestion medium volume ratios (i.e., 1:2.5; 1:5 or 1:10 w:v) and different digestion incubation times (i.e., 30, 60 or 120 min). Frequency of CD4+, CD8+, CD69+, CD103+, CD45RA+, CD27+, CCR7+, CD28+, KLRG1+, CD127+, PD1+, CXCR3+ and CXCR6+ T-cells (CD3+ cells) were quantified by flow cytometry in different tissue weight: digestion volume ratios (w:v) and different digestion incubation times (as indicated). Bars show mean value. Each dot represents data obtained from one individual. All groups were compared with each other and p values were calculated using Friedman test with Dunn’s multiple comparisons test. * p < 0.05. [file 12974_2022_2611_MOESM3_ESM.tif]

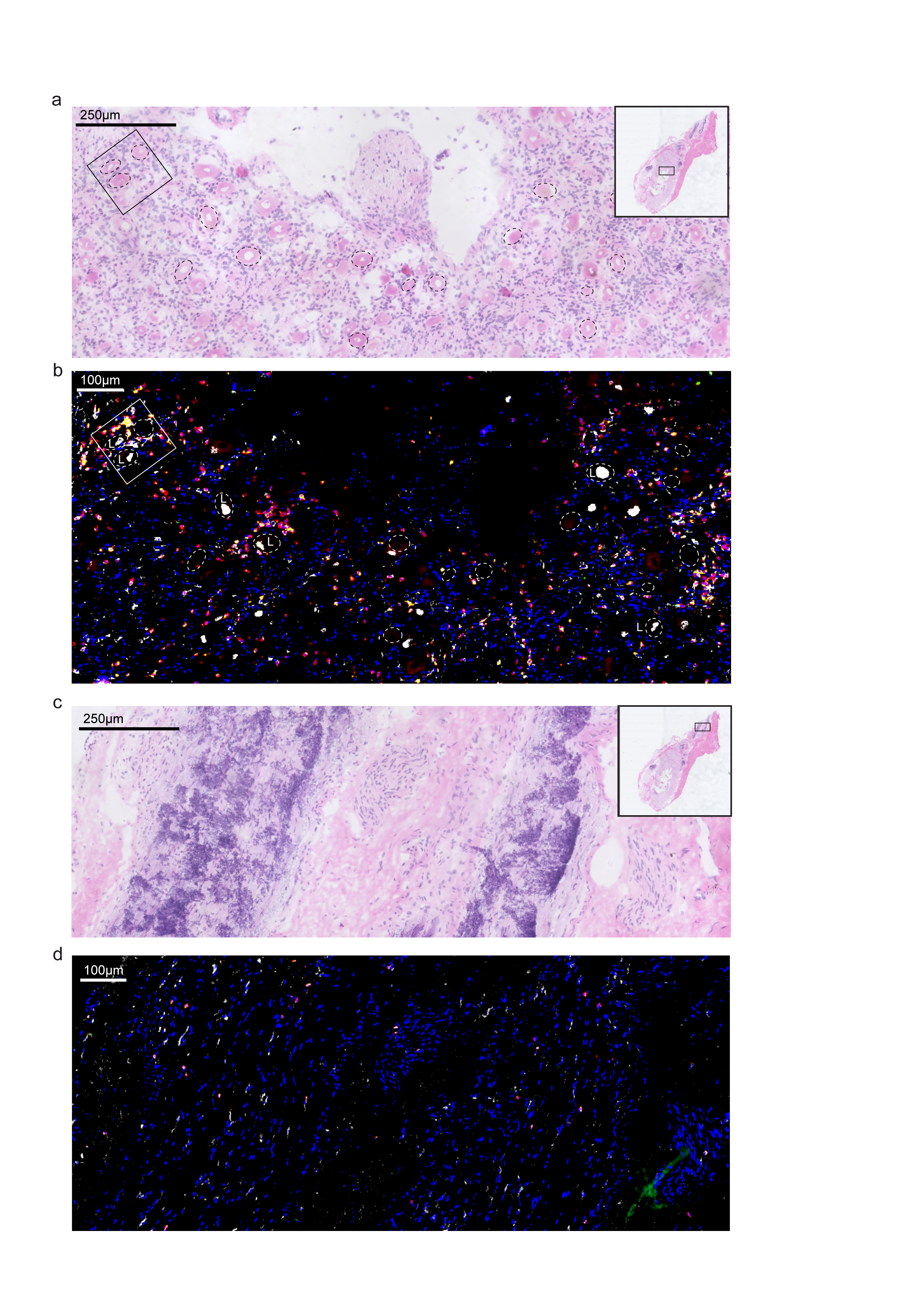

Supplement: Supplementary file 4 — Additional file 4: Figure S4. Localization of CD8 and CD4 T-cells within a latently HSV-1-infected human trigeminal ganglion. In situ analysis of a representative latently HSV-1-infected human trigeminal ganglion (TG), cut into serial 8 µm tissue sections, which was (1) conventionally stained with hematoxylin and eosin (a and c; insert shows whole TG section with rectangle region zoomed in for panels a and b, and c and d) and (2) triple immunofluorescence staining for CD3, CD4 and CD8 on a consecutive TG section (b and d). Central part of the TG containing neuronal somata, selected number of neuronal somata, with or without lipofuscin (L), are marked with a dotted circle (a and b), and peripheral region containing axon bundles and connective tissues but devoid of neuronal somata are shown (c and d). In panels b and d, T-cells (CD3+; red) are dispersed throughout the TG tissue, predominantly in the neuronal cell body TG region (panels a and b), where occasionally neuron-interacting T-cell clusters are found that are composed of both CD4 (white) and CD8 (green) T-cells (see squared region in panel b, which is presented in Fig. 5a). Majority of CD3+ cells co-expressed CD8 and nuclei were stained with DAPI (blue color). Original magnification was 5 × and scale bars illustrate size of the tissue section shown. [file 12974_2022_2611_MOESM4_ESM.tif]

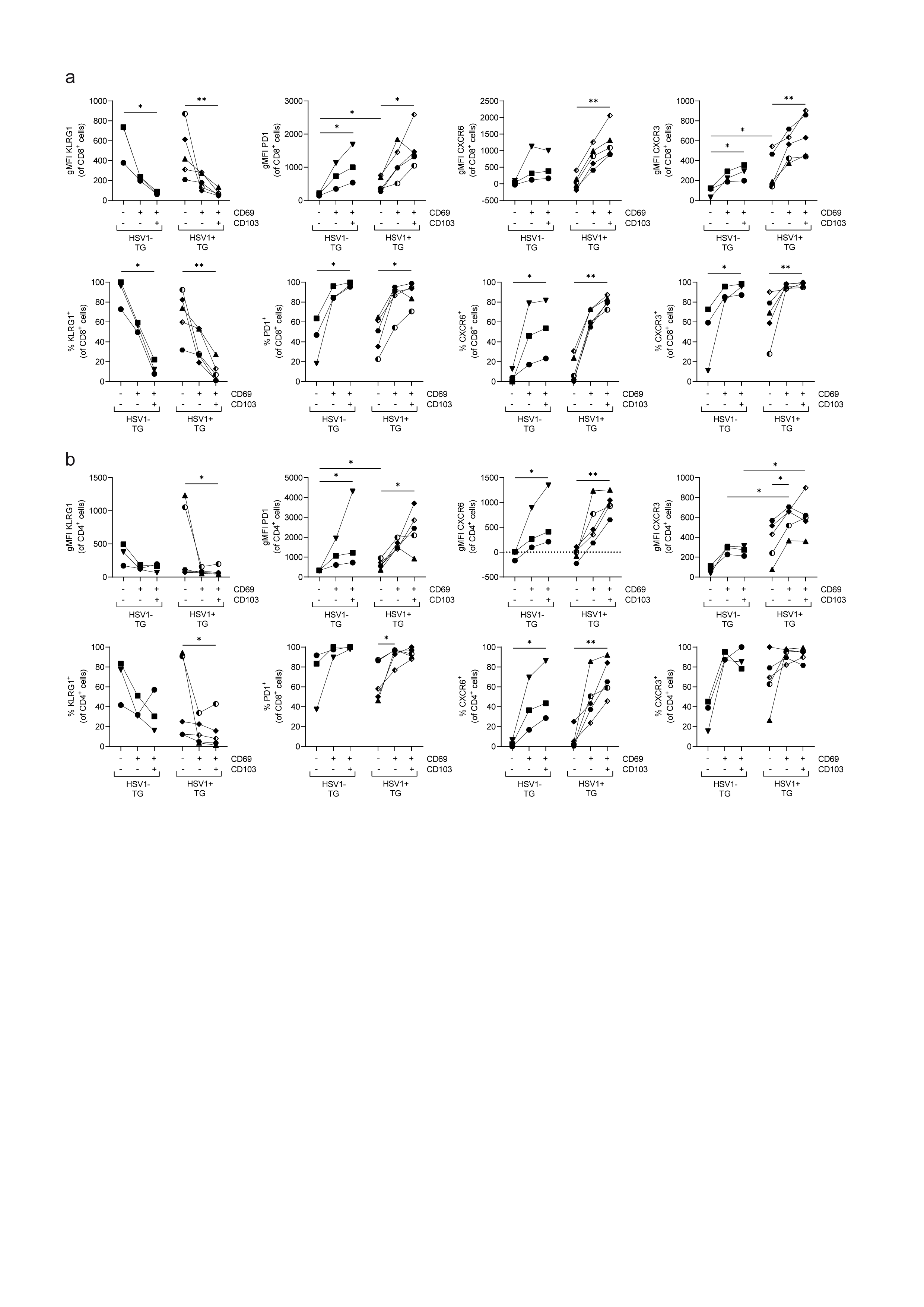

Supplement: Supplementary file 5 — Additional file 5: Figure S5. CD69+CD103+ T-cells recovered from human trigeminal ganglia seem phenotypically different from CD69+CD103- T-cells. Mononuclear cells were isolated from trigeminal ganglia (TG) of deceased brain donors (n = 8). Expression levels (geometric mean fluorescence intensity, gMFI) of KLRG1, PD1, CXCR6 and CXCR3 and percentages (%) of KLRG1+, PD1+, CXCR6+ and CXCR3+ CD8 (a) and CD4 (b) T-cells were quantified within the CD69 and CD103 subsets (as indicated) by flow cytometry. Each dot represents data obtained from one individual. P values were calculated using Friedman test with Dunn’s multiple comparisons test between CD69/CD103 subsets. Mann–Whitney test was performed to compare CD69/CD103 subsets in TG-derived CD8 and CD4 T-cells between HSV-1 naïve (HSV-1−) and latently HSV-1-infected individuals (HSV-1+). * p < 0.05 and ** p < 0.001. [file 12974_2022_2611_MOESM5_ESM.tif]
