# Supplementary material for: T-cells in human trigeminal ganglia express canonical tissue-resident memory T-cell markers
Source: J Neuroinflammation. 2022 Oct 6;19:249. doi: 10.1186/s12974-022-02611-x (PMC9535861; doi:10.1186/s12974-022-02611-x)
Supplement: Supplementary file 6 — Additional file 6: Table S1. Characteristics antibodies used in this study. [file 12974_2022_2611_MOESM6_ESM.docx]

| **Table S1. Characteristics antibodies used in this study.** | | | | |
| --- | --- | --- | --- | --- |
| **Antigen** | **Fluorochrome** | **Clone ID** | **Manufacturer** | **Application^*^** |
| CCR7 | BV650 | G043H7 | Biolegend | Flow cytometry |
| CD103 | BV711 | Ber-ACT8 | Biolegend | Flow cytometry |
| CD127 | PECF594 | HIL-7R-M21 | BD Biosciences | Flow cytometry |
| CD27 | BV510 | O323 | Biolegend | Flow cytometry |
| CD28 | BV605 | CD28.2 | BD Biosciences | Flow cytometry |
| CD3 | BUV661 | UCHT1 | BD Biosciences | Flow cytometry |
| CD4 | BUV737 | SK3 | BD Biosciences | Flow cytometry |
| CD45 | AF700 | 2D1 | Biolegend | Flow cytometry |
| CD45RA | BUV563 | HI100 | BD Biosciences | Flow cytometry |
| CD69 | BUV395 | FN50 | BD Biosciences | Flow cytometry |
| CD8 | BUV805 | SK1 | BD Biosciences | Flow cytometry |
| CXCR3 | BV421 | G025H7 | Biolegend | Flow cytometry |
| CXCR6 | APC | K041E5 | Biolegend | Flow cytometry |
| KLRG1 | AF488 | 13F12F2 | Bioscience | Flow cytometry |
| PD1 | BB700 | EH12.1 | BD Biosciences | Flow cytometry |
| TCRγδ | APCFire750 | B1 | Biolegend | Flow cytometry |
| CD3ε | None | SP7 | ThermoScientific | *In situ* (= all) |
| CD4 | None | 4B12 | Dako | *In situ* (= 1) |
| CD8αβ | None | YTC182.20 | Bio-Rad | *In situ* (= 1) |
| CD69 | None | FN50 | Biolegend | *In situ* (= 2) |
| CD103 | None | 2G5-1 | ThermoScientific | *In situ* (= 2) |
| CD137 | None | 4B4-1 | BD Biosciences | *In situ* (= 3) |
| CD40L | None | Polyclonal goat | R&D | *In situ* (= 3) |
| p16INK4a | None | 5A8A4 | Sigma Aldrich | *In situ* (= 4) |
| PD1 | None | EH33 | Cell Signaling Technologies | *In situ* (= 4) |
| TIA-1 | None | 2G9A10F5 | Beckman Coulter | *In situ* (= 5) |
| CD107a | None | 1D4B | Invitrogen | *In situ* (= 5) |
| Ki-67 | None | MIB-1 | Dako | *In situ* (= 6) |
| Rabbit IgG | AF594 | Polyclonal goat | ThermoScientific | *In situ* (= 1, 5 and 6) |
| Mouse IgG1 | AF647 | Polyclonal goat | ThermoScientific | *In situ* (= 1, 5, and 6) |
| Rat IgG | AF488 | Polyclonal goat | ThermoScientific | *In situ* (= 1 and 5) |
| Rabbit IgG | AF647 | Polyclonal chicken | ThermoScientific | *In situ* (= 2 and 4) |
| Mouse IgG1 | AF488 | Polyclonal goat | ThermoScientific | *In situ* (= 2 and 4) |
| Mouse IgG2a | AF594 | Polyclonal goat | ThermoScientific | *In situ* (= 2 and 4) |
| Rabbit IgG | AF594 | Polyclonal chicken | ThermoScientific | *In situ* (= 3) |
| Mouse IgG | AF647 | Polyclonal chicken | ThermoScientific | *In situ* (= 3) |
| Goat IgG | AF488 | Polyclonal donkey | ThermoScientific | *In situ* (= 3) |

^*^ Numbers between brackets indicate combination primary and secondary antibodies used in the six panels of triple immunofluorescent *in situ* analysis of consecutive human trigeminal ganglia tissue sections.
