# Supplementary material for: T-cells in human trigeminal ganglia express canonical tissue-resident memory T-cell markers
Source: J Neuroinflammation. 2022 Oct 6;19:249. doi: 10.1186/s12974-022-02611-x (PMC9535861; doi:10.1186/s12974-022-02611-x)
Supplement: Supplementary file 7 — Additional file 7: Table S2. Markers of human T-cell differentiation. [file 12974_2022_2611_MOESM7_ESM.docx]

**Table S2. Markers of human T-cell differentiation^*^.**

| **Marker** | **T_NA_** | **T_CM_** | **T_EM_** | **T_EMRA_** | **T_RM_** |
| --- | --- | --- | --- | --- | --- |
| CD45RA | + | - | - | + | - |
| CD27 | + | + | -/+ | -/+ | -/+ |
| CD28 | + | + | -/+ | -/+ | -/+ |
| CCR7 | + | + | - | - | - |
| CD127 | + | + | + | -/+ | -/+ |
| KLRG-1 | - | + | + | + | + |
| PD1 | - | + | + | + | + |
| CXCR3 | - | + | + | + | + |
| CD69 | - | - | - | - | + |
| CD103 | - | - | - | - | -/+ |
| CXCR6 |  |  |  |  | + |

T_NA_, naïve T-cell; T_CM_, central memory T-cell; T_EM_, effector memory T-cell, T_EMRA_, terminally

differentiated effector memory T-cells and T_RM_, tissue-resident T-cell. ND, not determined.

Marker expression adapted from following references: 1, 30 and 31.
